# A highly stable full-polymer electrochemical deionization system: dopant engineering & mechanism study†

**DOI:** 10.1039/d4mh00494a

**Published:** 2024-06-18

**Authors:** Yi-Heng Tu, Hung-Yi Huang, Yu-Hsiang Yang, Louis C. P. M. de Smet, Chi-Chang Hu

**Affiliations:** a Department of Chemical Engineering, National Tsing Hua University 101, Section 2, Kuang-Fu Road Hsin-Chu 300044 Taiwan cchu@che.nthu.edu.tw https://www.cchulecam.com/ +886-3-5736027 +886-3-5736027; b Advanced Interfaces & Materials, Laboratory of Organic Chemistry, Wageningen University Stippeneng 4 Wageningen 6708 WE The Netherlands louis.desmet@wur.nl https://www.louisdesmet.nl

## Abstract

Electrochemical deionization (ECDI) has emerged as a promising technology for water treatment, with faradaic ECDI systems garnering significant attention due to their enhanced performance potential. This study focuses on the development of a highly stable and efficient, full-polymer (polypyrrole, PPy) ECDI system based on two key strategies. Firstly, dopant engineering, involving the design of dopants with a high charge/molecular weight (*M*_W_) ratio and structural complexity, facilitating their effective integration into the polymer backbone. This ensures sustained contribution of strong negative charges, enhancing system performance, while the bulky dopant structure promotes stability during extended operation cycles. Secondly, operating the system with well-balanced charges between deionization and concentration processes significantly reduces irreversible reactions on the polymer, thereby mitigating dopant leakage. Implementing these strategies, the PPy(PSS)//PPy(ClO_4_) (PSS: polystyrene sulfonate) system achieves a high salt removal capacity (SRC) of 48 mg g^−1^, an ultra-low energy consumption (EC) of 0.167 kW h kg_NaCl_^−1^, and remarkable stability, with 96% SRC retention after 104 cycles of operation. Additionally, this study provides a detailed degradation mechanism based on pre- and post-cycling analyses, offering valuable insights for the construction of highly stable ECDI systems with superior performance in water treatment applications.

## Abstract 2

Dopants with high charge density and structural complexity, combined with balanced operating conditions, can significantly enhance the performance and stability of a full-polymer ECDI system.

## Graphical abstract

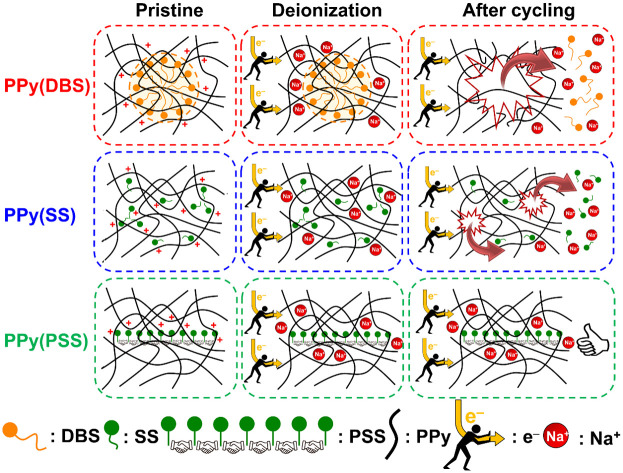

**New concepts**

In this manuscript, we present a novel approach for utilizing conducting polymers (polypyrrole), through dopant engineering and mechanistic exploration. Our study identifies dopant leakage as the primary factor of performance decline in polypyrrole within electrochemical deionization (ECDI) systems during extended operational cycles. To address this challenge, we propose two pivotal strategies. Firstly, employing dopants with high charge-to-molecular-weight ratios amplifies the charge density within a confined volume, thereby enhancing performance. Secondly, utilizing dopants with significant structural complexity facilitates their effective integration into the polymer matrix, preventing leakage and enhancing stability. We illustrate these concepts using dopants with varied charge-to-*M*_W_ ratios (*e.g.*, PSS (polystyrene sulfonate), SS (styrene sulfonate), and DBS (dodecyl benzene sulfonate)) and dopants with equivalent charge-to-*M*_W_ ratios but differing structural complexities (PSS *vs.* SS). Additionally, our approach emphasizes maintaining a balanced charge during system operation to minimize irreversible reactions like dopant leakage. By implementing these strategies, we significantly improve the stability of polypyrrole, addressing a common concern in its applications. This concept offers a fresh perspective for leveraging polypyrrole and dopant design across diverse electrochemical domains, contributing to excellent stability for practical applications. These insights are crucial not only for ECDI technology but also for energy and electrochromic applications, appealing to a broad readership.

## 1. Introduction

Electrochemical deionization (ECDI) is an emerging technology with applications of desalination and targeted ion recycling.^1^ The fundamental principle of ECDI involves the removal of ions from a solution using electricity through either the electric double layer (EDL) mechanism or faradaic reactions. ECDI has the potential to replace or complement traditional purification methods such as distillation, multi-stage flash, and reverse osmosis (RO) due to its low energy consumption, ease of operation, and adaptability across a wide range of applications, particularly when dealing with brackish water, small-scale implementations and/or ion-selective separations.^2,3^ Research efforts in the field of ECDI are steadily expanding, particularly in the development of new materials.

Drawing inspiration from batteries, supercapacitors, and various electrochemical techniques,^4–6^ researchers have introduced numerous materials into the field of ECDI. These materials can be broadly categorized into two groups: (1) porous materials that form EDLs to store ions, and (2) materials that leverage faradaic reactions to capture ions. Typical examples of porous materials include carbon materials and their derivatives,^7–10^ known for their low cost and ease of control over working conditions. However, EDL materials often exhibit a low salt removal capacity and poor selectivity toward ions, limiting their range of applications. In contrast, faradaic materials have gained increasing attention in recent years due to their high removal capacity and rapid ion capture rates. Furthermore, through thoughtful designs, faradaic materials can also demonstrate outstanding ion selectivity towards specific ions.^11–16^ Common faradaic materials include metal oxides,^17–19^ metals,^20^ conducting polymers,^21,22^ Prussian Blue analogs (PBAs),^23–26^ Mxene,^27,28^ covalent organic frameworks (COFs),^29–31^ organo-metallic compounds,^32^ metal organic frameworks (MOFs),^33^ and their derivatives or hybrid materials.^34–37^

Conducting polymers, also known as intrinsically conducting polymers (ICPs), are organic polymers with the ability to conduct electricity.^38,39^ These polymers have attracted significant attention due to their favorable electrochemical properties and ease of fabrication, making them the subject of extensive research for decades. They find applications in a wide range of fields, including energy,^40–42^ environmental sciences,^43,44^ and various electricity-related industries. Polypyrrole (PPy) is a typical and widely used ICP that has been studied for decades. The synthesis of PPy is easily achieved through either chemical oxidation methods^45^ or electrochemical polymerization.^18,22^ What adds to its intrigue is that PPy can exhibit a range of distinct characteristics by simply altering the dopants used during the synthesis process.^46–48^ For instance, leveraging the size effect of dopants, when PPy is doped with large molecules, such as *p*-toluene sulfonate (*p*-TS) or dodecylbenzene sulfonate (DBS), the resulting polymer films demonstrate a cation-exchange ability.^21,22^ This occurs because the large dopants are prone to being trapped within the polymer backbone during synthesis, generating permanent negative charges residing within the PPy structure, which in turn attract cations. Conversely, when PPy is doped with small molecules, such as chloride or perchlorate, the polymer films can exhibit an anion-exchange ability. This is due to the relatively high mobility of these small anions within the polymer backbone, allowing them to exchange with anions in the solution.^18^

Due to its dual characteristic of acting as both cation- and anion-exchange materials, PPy is extensively utilized as the active material in ECDI systems because the utilization of the same redox reactions can reduce the ECDI cell voltage during the ion capturing and releasing process. Furthermore, a comprehensive polymer system can be constructed by employing PPy with different dopants as positive and negative electrodes. This system exhibits numerous advantages, including a high salt removal capacity (SRC), a medium to high salt removal rate (SRR), and a low energy consumption. For instance, in our previous work, we successfully developed a full polymer system using *p*-TS-doped PPy for cation capturing and perchlorate-doped PPy for anion capturing. This system achieved over 70 mg g^−1^ of SRC and 0.04 mg g^−1^ s^−1^ of SRR in a 40 mM NaCl solution, with low energy consumption of around 0.2 kW h kg_NaCl_^−1^.^21^ Additionally, this PPy system demonstrates another dual functionality, serving both in ion-recycling and ion-concentrating without the need for a membrane. This phenomenon can be attributed to the compelling memory effect that allows PPy to retain its states even after the applied current is interrupted.^17^ All the above viewpoints make this polymer system a potential candidate for practical applications in the real world. However, when contemplating the potential for commercialization, the stability of the system emerges as one of the most critical factors for reducing operational costs to effectively competing with other mature purification techniques.

Therefore, the objective of this research is to pursue a highly stable and energy-efficient, full-PPy ECDI system without compromising its SRC through a mechanistic study of ion-capturing and material degradation. By selecting appropriate dopants and optimizing the operational parameters, this full-PPy ECDI system demonstrates 96% of SRC retention after 104 cycles (approximately 70 hours) with an SRC of 48 mg g^−1^ and a very low energy consumption of 0.16 kW h kg_NaCl_^−1^. Furthermore, a degradation model of PPy was proposed, offering a general guideline for designing ICP-based systems. This research provides valuable insights for the application of ICPs in the electrochemical-related field, pushing the potential of full-polymer systems to new heights.

## 2. Experimental

### 2.1. Preparation of titanium current collectors

The preparation of titanium current collectors largely followed our previous work.^18^ The only variation lies in the shape of the titanium sheet, tailored to accommodate different ECDI cells. The detailed procedure is described in the ESI.†

### 2.2. Synthesis of electroactive materials

The PPy film in this research was synthesized through electrochemical polymerization. A two-electrode system, with a pretreated titanium sheet as the anode and platinum wire as the cathode, was employed to deposit the electroactive materials. The electrolyte solution was prepared by dissolving 0.1 M of pyrrole monomer and 0.05 M of dopants, including sodium styrene sulfonate (SS), sodium dodecylbenzene-sulfonate (DBS), poly(sodium 4-styrenensulfonate) (PSS) with an average molecular weight (*M*_W_) of ∼70 000, and lithium perchlorate (LiClO_4_ as a source for ClO_4_^−^, hereafter referred to as ClO_4_ when used in a polymer system) in deionized (DI) water. A constant current with a current density of 5 mA cm^−2^ was applied to the system for 1200 seconds. Subsequently, the PPy electrode was washed with DI water and stored in DI water before undergoing further analysis or deionization tests. The synthesized PPy electrodes were named PPy(SS), PPy(DBS), PPy(PSS), and PPy(ClO_4_).

### 2.3. Material characterization

The pristine PPy electrodes were investigated using several material characterization techniques to understand their basic properties. A field-emission scanning electron microscope (FESEM, Hitachi SU-8010) was utilized to study the surface morphology. The surface composition and chemical environment were examined through high-resolution X-ray photoelectron spectroscopy (HRXPS, ULVAC-PHI, PHI Quantera II). The internal structure of PPy was observed through X-ray diffraction (XRD, Bruker, D8 Advance Eco). Electrochemical characteristics were measured in a three-electrode system consisting of a platinum wire as the counter electrode and a commercial Ag/AgCl (3 M KCl) as the reference electrode, with a 10 mM NaCl solution as the electrolyte. Electrochemical tests were conducted using both an Autolab (Metrohm) and a CHI 6273e electrochemical workstation (CH Instruments).

The PPy electrodes underwent post-cycling analysis after completing 100 cycles in a full ECDI cell, which included SEM, XPS, and electrochemical analysis under identical conditions.

### 2.4. Deionization tests

The deionization tests were undertaken in a circulation system which completely followed our previous work^18^ with a reduction in the total solution volume from 75 mL to 55 mL to increase the resolution of the data. A scheme of the entire desalination test is shown in Fig. S1 (ESI†).

The operational parameters were determined on the basis of basic electrochemical characterization. During the desalination test, the conductivity value and electrochemical profile were recorded and subjected to further analysis by converting them into multiple performance indicators. The first performance indicator, salt removal capacity (SRC, *Γ*), which signifies the deionization capability of the ECDI cell, was calculated using the following equation:1
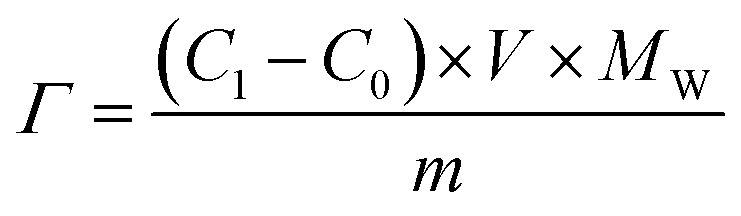
where *C*_1_ is the salt concentration at a certain time, *C*_0_ is the initial salt concentration, *V* represents the solution volume cycled in the system, *M*_W_ is the molecular weight of NaCl (58.44 g mol^−1^), and *m* indicates the total mass of all active materials on both positive and negative electrodes. Secondly, the salt removal rate (SRR), which indicates how quickly the ECDI system can capture salt from the solution, was calculated using the following equation:2
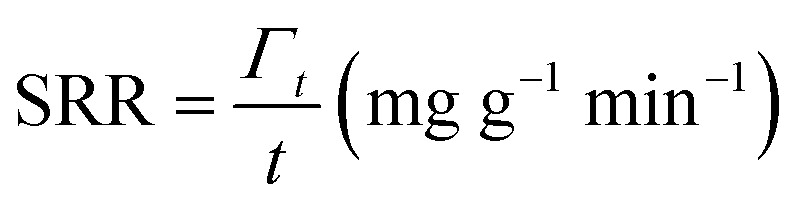
where *Γ*_*t*_ is the SRC at the discharging time equal to *t*. The retention of salt removal capacity (*R*_SRC_) represents the stability of the system during repeated operation, serving as a crucial indicator for potential future commercialization.3
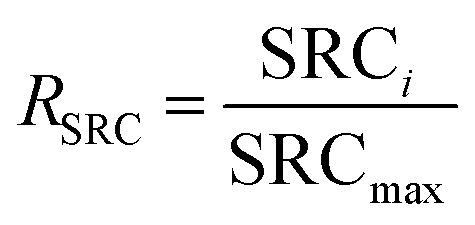
where SRC_*i*_ represents the specific SRC (mg g^−1^) of the *i*th cycle and SRC_max_ (mg g^−1^) is the maximum SRC. The last indicator is the energy consumption (EC), which reflects the energy efficiency of the entire system.4
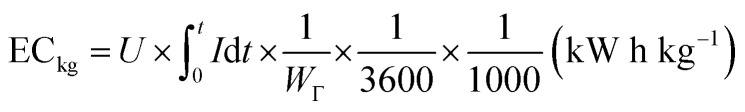
where EC is the energy consumption, *I* indicates the current responses of the system (A), and *U* represents the externally applied voltage (V) under the charging state or the difference in the open-circuit potentials between the positive and negative electrodes under the discharging state. Integrating *I* over time (s) expresses the total charges consumed by the system (Coulomb). *W*_*Γ*_ is the mass of the salts (kg) removed by the system during the operation period *t* (s).

## 3. Results & discussion

### 3.1. Material characterization

In Fig. S2(a) (ESI†), we present the calibration curve of mass loading *versus* charges for PPy films with various dopants. Notably, all results display a linear relationship, indicating effective control of mass loading through the constant current electrochemical polymerization method. The disparity in slope is attributed to the *M*_W_ of dopants, as evidenced by the correlation plotted in Fig. S2(b) (ESI†) between slope and *M*_W_ per negative charge. Interestingly, dopants carrying a single negative charge per unit (DBS, SS, and ClO_4_) exhibit a perfect linear relationship, whereas PSS, with a substantial number of negative charges in a single unit, shows a noticeable drop in slope. This phenomenon suggests that the PPy systems using DBS, SS, and ClO_4_ as dopants exhibit very similar amounts of trapped dopants in the polymer matrix. In contrast, the bulky structure of PSS may impede the electrochemical polymerization process, resulting in fewer trapped dopants and reduced mass loading. In this study, the mass loading of PPy(PSS), PPy(SS), PPy(DBS), and PPy(ClO_4_) is 2.5 mg cm^−2^, 3.23 mg cm^−2^, 4.76 mg cm^−2^, and 2.37 mg cm^−2^, respectively.

**Fig. 1:**
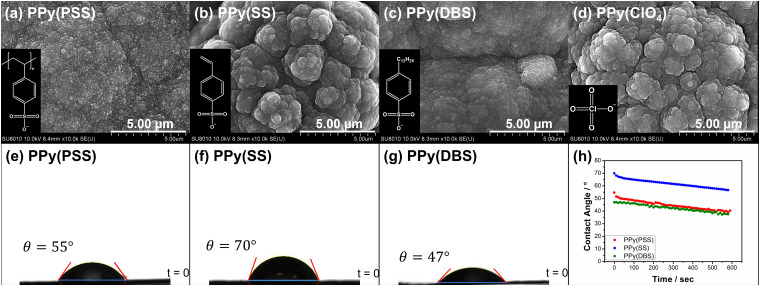
SEM photographs showing the surface morphologies of (a) PPy(PSS), (b) PPy(SS), (c) PPy(DBS), and (d) PPy(ClO_4_) under 10k magnification, with the structures of dopants showcased in the bottom left corner. Contact angle analysis of (e) PPy(PSS), (f) PPy(SS), and (g) PPy(DBS). (h) Time profiles of contact angle analysis.

Fig. 1(a)–(d) showcase the surface morphologies of PPy(PSS), PPy(SS), PPy(DBS), and PPy(ClO_4_) as investigated with FESEM. All samples display a cauliflower-like structure, characteristic of electrochemically polymerized PPy.^49^ However, the surface morphologies of PPy(SS) and PPy(ClO_4_) reveal larger grains and more pronounced aggregation compared to the other two samples. This observation suggests that aggregation occurs more readily when the dopants are smaller in size (dopant size: PSS > DBS > SS > ClO_4_), resulting in grain formation on the surface.^50^ Contact angle analysis results using the 10 mM NaCl solution align with the SEM findings. Materials with less aggregation demonstrate better hydrophilicity, with contact angles ranging between 45° and 55°, as shown in Fig. 1(e) and (g). Conversely, the contact angle increases to 70° for PPy(SS) (Fig. 1(f)), indicating more aggregate particles on the surface. Additionally, PPy(DBS) exhibits superior hydrophilicity due to its strong surfactant characteristic, leading to uniform monomer dispersion and micelle formation during polymerization, resulting in a smooth surface. It is noteworthy that the PPy(ClO_4_) electrode appeared very fragile after drying, resulting in severe cracking, making it difficult to assess its contact angle properties. Fig. 1(h) illustrates the contact angle profile over time, all samples show a 10-degree decrease after 10 minutes, suggesting similar polymer backbone structures contributing to their analogous behavior.

**Fig. 2:**
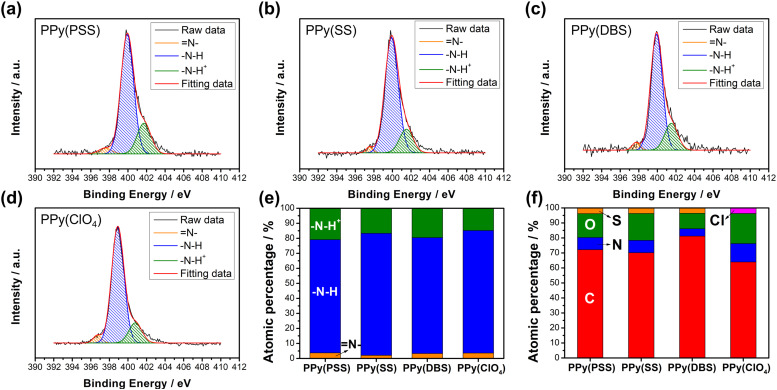
HRXPS N 1s core level spectra for (a) PPy(PSS), (b) PPy(SS), (c) PPy(DBS), and (d) PPy(ClO_4_). (e) Atomic percentages of different nitrogen species and (f) the atomic percentages of S, N, O, C, and Cl on the surface for four PPy materials.

Fig. S3 (ESI†) provides a magnified view (100k) of the SEM results, revealing details that further confirm similar results as in Fig. 1 that the PPy particles aggregate into larger grains on the surface of PPy(SS) and PPy(ClO_4_). Fig. S4 (ESI†) presents the surface composition of all four samples through X-ray photoelectron spectroscopy (XPS) analysis. Four elements on the surface of these samples were detected: C, N, O, and Cl. High-resolution analysis at the N 1s core level offers more details about the electrode surface, as shown in Fig. 2(a)–(d). The strongest peak at 399.9 eV (398.9 eV for PPy(ClO_4_)) indicates the presence of neutral nitrogen in the PPy backbone (–N–H bond).^51,52^ Additionally, a peak shifted to the higher binding energy around 401.5 to 401.7 eV (400.8 eV for PPy(ClO_4_)) is assigned to the positively charged nitrogen structure (–N–H^+^ bond),^53^ while a peak at the lower binding energy around 397.5 to 397.7 eV (396.7 eV for PPy(ClO_4_)) corresponds to the imine structure in the neutral pyrrole ring (=N– bond).^54^ In Fig. 2(e), all four samples exhibit a similar composition at the N 1s core level. Approximately 80–85% of the nitrogen content can be attributed to neutral nitrogen in the pyrrole ring (–N–H and =N– bond), with 15–20% representing positively charged nitrogen (–N–H^+^ bond) which may be formed during the electrochemical polymerization (oxidation) process. Fig. 2(f) presents the atomic percentage of each element determined through high-resolution XPS analysis. The doping degree of each electrode was determined by analyzing the ratio between sulfate (or chloride) and nitrogen. Specifically, by dividing the number of dopants (sulfate for PSS, DBS, SS, and chloride for ClO_4_) with the number of pyrrole rings (nitrogen), the doping ratio can be calculated. Consequently, the doping ratios for PPy(PSS), PPy(SS), PPy(DBS), and PPy(ClO_4_) were found to be 0.46, 0.45, 0.73, and 0.3, respectively. DBS exhibits the highest doping ratio due to its exceptional surfactant characteristics, enabling the formation of well-dispersed micelles in the electrolyte and incorporation into the polymer backbone during polymerization. Additionally, SS and PSS show similar and relatively high doping ratios, attributed to their comparable charge/*M*_W_ ratio. It is noteworthy that the doping ratio of ClO_4_ is the lowest, possibly due to the size of ClO_4_, which increases the mobility of the dopants, making them more challenging to trap. The doping ratio aligns with the XRD results shown in Fig. S5 (ESI†), where the peak located at the lower degree between 10° to 30° indicates the interspacing between polymer chains induced by the dopants.^22,55^ The PPy(DBS), with the largest size and highest doping ratio, coupled with micelle formation during polymerization,^55,56^ demonstrates the largest *d*-spacing of *θ* = 18°, 4.9 Å. The PPy(SS) demonstrates the second highest doping ratio and a larger size compared to ClO_4_. Additionally, it has the potential to form a micelle-like structure, leading to a medium *d*-spacing of *θ* = 20°, 4.4 Å. Lastly, the PPy(PSS) with no micelle formation shows the smallest *d*-spacing of *θ* = 21°, 4.2 Å. The second peak around *θ* = 26° indicates the inter-counterion interaction, which is similar among all samples.^57^

**Fig. 3:**
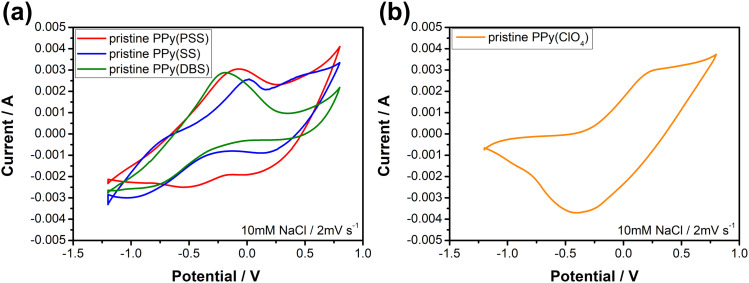
Cyclic voltammograms of (a) positive electrode materials, PPy(PSS), PPy(SS), and PPy(DBS) and (b) a negative electrode material, PPy(ClO_4_) in the 10 mM NaCl solution with a scan rate of 2 mV s^−1^; the potentials are against Ag/AgCl.

Fig. 3 depicts the cyclic voltammetric results for the PPy materials in a 10 mM NaCl solution at a scan rate of 2 mV s^−1^. The materials used as positive electrodes (for sodium-capturing) are presented in Fig. 3(a). An evident oxidation peak is observed at a high potential range (>0.6 V *vs.* Ag/AgCl), indicating an irreversible reaction that could probably damage the electrode.^58^ Therefore, it is recommended to control the upper potential limit for PPy below 0.6 V. Another notable oxidation peak, ranging from −0.3 V to 0 V, indicates the oxidation of the polymer backbone, resulting in the expulsion of cations from the polymer. Conversely, the reduction peak at −0.5 V for PPy(PSS) and −0.8 V to −1 V for PPy(SS) and PPy(DBS) can be attributed to the insertion of cations. Additionally, mild peaks observed in the CV curve, such as the 0.1 V reduction for PPy(PSS) and the −0.8 V oxidation/0.25 V reduction for PPy(SS), likely correspond to the movement of anions in the system. The CV curve of PPy(ClO_4_) (Fig. 3(b)) exhibits a distinct and broad reduction peak located at −0.4 V, indicating the ejection of anions from the polymer backbone. Due to the small size of ClO_4_^−^ dopants, they are easily expelled and exchanged with other anions in the solution during the oxidation process. The oxidation peak observed at 0.25 V can be attributed to the re-entry of anions. The specific capacitance for pristine materials was calculated using CVs, serving as an indicator to assess electrode degradation. Detailed values and methods are provided in Table S1 (ESI†).

### 3.2. Deionization tests

**Fig. 4:**
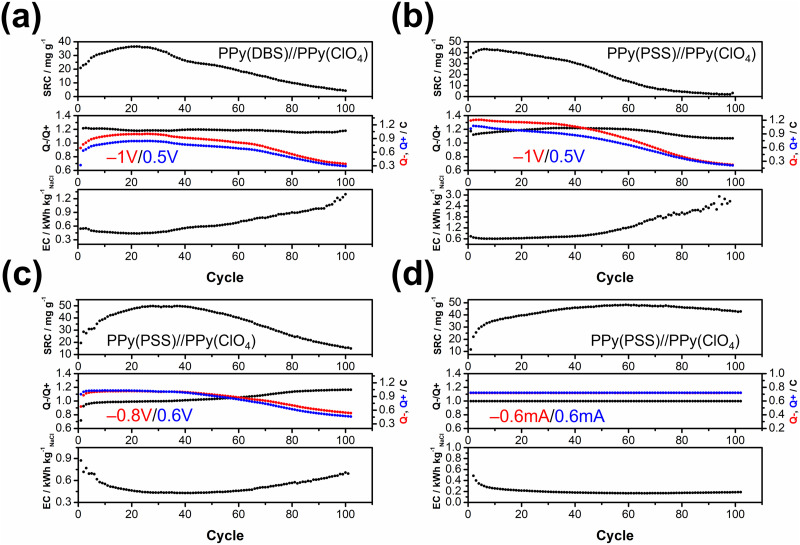
Salt removal capacity (SRC) profile, *Q*−/*Q*+ value, and energy consumption (EC) profile for (a) PPy(DBS)//PPy(ClO_4_) with deionization, concentration = −1 V/0.5 V, 20 min/20 min, (b) PPy(PSS)//PPy(ClO_4_) with deionization, concentration = −1 V/0.5 V, 20 min/20 min, (c) PPy(PSS)//PPy(ClO_4_) with deionization, concentration = −0.8 V/0.6 V, 20 min/20 min, and (d) PPy(PSS)//PPy(ClO_4_) with deionization, concentration = 0.6 mA/0.6 mA, 20 min/20 min.

The objective of the deionization tests is to elucidate the relationship between dopants, operating parameters, and system performance (SRC, energy consumption, stability) to facilitate a more comprehensive understanding of the underlying mechanism. Through the analysis of these results, our aim is to establish a set of general principles for designing similar full-polymer systems. To begin, based on literature findings, DBS emerged as the most commonly used and effective dopant for cation removal when combined with PPy.^59,60^ Thus, the deionization process commenced with the composition of PPy(DBS)//PPy(ClO_4_). The determination of the working potential window was guided by the CV curve, with the reduction peak of PPy(DBS) occurring around −0.8 V and the oxidation peak of PPy(ClO_4_) located at approximately 0.2 V *vs.* Ag/AgCl. Consequently, the potential for deionization was set at −1 V. Conversely, the potential for the concentration process was initially set at 0.3 V (−0.2 V for PPy(DBS) and −0.5 V for PPy(ClO_4_)), which was subsequently raised to 0.5 V due to insufficient ion repulsion during the experiment. The results for the PPy(DBS)//PPy(ClO_4_) system with deionization/concentration at −1 V/0.5 V, 20 min/20 min, are depicted in Fig. 4(a), and the detailed values are listed in Table S2 (ESI†). The SRC reaches its peak value of 36 mg g^−1^ around cycle 20, stabilizes until cycle 30, and then starts to decline. Note that the increase of SRC value of the first few cycles can be recognized as the activation process, and the speed of this process is mainly attributed to the amount of applied charges (*Q*− and *Q*+), in which larger applied charges result in reaching its peak value in a shorter time. After 100 cycles of operation, the remaining SRC is only 4 mg g^−1^ (11.5%), indicating the poor stability of this setup. The energy consumption (EC) value was also monitored and consistently reflected the SRC trend. The lowest EC value was recorded as 0.43 kW h kg_NaCl_^−1^, increasing to over 1 kW h kg_NaCl_^−1^ at the last cycle. The ratio between charges during the deionization and concentration processes (*Q*−/*Q*+) was also recorded to serve as an indicator for performance improvement. For this system, the *Q*−/*Q*+ ratio remained at 1.2 throughout the process. However, the total amount of charges for the deionization (red) and concentration (blue) processes decreased from their highest point of 0.9 to 0.3 after 100 cycles. To further explore the origin for this dramatic degradation in the SRC, we conducted post-cycling CV analysis for both electrodes. Fig. S6(a) (ESI†) (detailed values are presented in Table S3, ESI†) reveals an 84% loss in specific capacitance for PPy(DBS) and a 43% loss for PPy(ClO_4_). In other words, the degradation of the SRC in this system can mainly be attributed to the failure of PPy(DBS). This phenomenon can be related to the doping mechanism of DBS during the fabrication of the PPy film. As DBS is a potent surfactant, it tends to form micelles in the aqueous solution, with the hydrophilic head forming the outer layer and the hydrophobic tail forming the inner core.^56,61^ The critical micelle concentration is around 10 mM in pyrrole solution. In some cases, DBS forms cylindrical micelles with even larger sizes.^56,62^ These large-sized micelles become trapped in the PPy backbone during the polymerization process, providing negative charges within the structure. Although most of the DBS remains relatively immobile within the structure, a small portion is expelled from the polymer backbone during electrochemical processes. This expulsion is evidenced by the ICP-OES results in the further discussion. Due to the large size of DBS micelles, its removal damages the entire polymer structure and causes PPy to lose its negative charges, resulting in a significant decline in specific capacitance and salt removal capacity.

To address this issue, we propose two potential methods for improvement. The first involves dopant engineering, where dopants that are even more immobile are designed and utilized to pursue better performance. The second method aims to reduce the driving force for expelling these dopants, thus stabilizing the structure throughout the entire process through optimization of operational parameters. When considering dopant engineering, two factors determine the performance of a dopant. Firstly, charge/*M*_W_ ratio (charge density) plays a crucial role. If a dopant can offer the same number of negative charges within a smaller structure or with a lower molecular weight, it should yield a higher SRC value due to its stronger attraction to the target ions. The second factor is the bulkiness of the dopant, which is essential for preventing dopant leakage during operation. However, there is typically a tradeoff between bulkiness and charge/*M*_W_ ratio (charge density), as bulky dopants tend to have larger sizes. The optimal solution, balancing both factors, involves linking smaller dopants with higher charge density to form a bulky structure. Polystyrene sulfonate (PSS) emerges as a promising candidate due to the larger charge density of each monomer compared to DBS, coupled with its inherently bulky polymer structure. Fig. 4(b) presents the results for PPy(PSS)//PPy(ClO_4_) under the same operating parameters as Fig. 4(a). The system achieves the highest SRC of 43 mg g^−1^, yet experiences a sharp degradation after 40 cycles. Additionally, the *Q*−/*Q*+ ratio remains around 1.1–1.2, similar to the system using DBS. However, the EC value is higher in the first 40 cycles (∼0.6 kW h kg_NaCl_^−1^) and increases dramatically thereafter. Although the performance of the PPy(PSS)//PPy(ClO_4_) system does not appear significantly better compared to the PPy(DBS)//PPy(ClO_4_) system, an intriguing phenomenon was observed in the post-cycling CV analysis shown in Fig. S6(c) (ESI†). The specific capacitance for PPy(PSS) exhibits only a 0.5% decay, indicating remarkable stability of the PPy(PSS) structure. This result aligns with the assumptions made in the dopant engineering part, underscoring how the higher charge density and bulkiness contribute to both higher SRC values and electrode stability. However, in this case, degradation primarily occurs at the PPy(ClO_4_) side, likely due to unbalanced charges triggering side reactions and damaging the electro-active materials.

In our exploration of methods to improve system stability, we introduce a second approach involving the balancing of charges between deionization and concentration processes. Excessive charges can lead to various issues harmful to system performance and stability, including dopant leakage and irreversible side reactions. To address these concerns, we advocate for the implementation of operating parameters characterized by well-balanced charges. Previous experiments revealed an imbalance (Fig. 4(a) and (b)), where more charges were applied during deionization than during concentration, creating a situation where dopants were expelled at the positive side and inducing over-oxidation at the negative side. To address this, we adjusted the applied potential for deionization and concentration from −1 V/0.5 V to −0.8 V/0.6 V, as depicted in Fig. 4(c). During the initial 40 cycles, the *Q*−/*Q*+ ratio closely approached 1. This balanced charge distribution corresponded to an increase in the SRC, achieving the highest SRC and SRR of 50 mg g^−1^ and 2.5 mg g^−1^ min^−1^, respectively. Furthermore, this adjustment resulted in a reduced energy consumption (EC) value, averaging around 0.43 kW h kg_NaCl_^−1^, which improved upon previous results. Subsequent post-cycling CV analyses in Fig. S6(e) and (f) (ESI†) revealed moderate capacitance (*C*_P_) losses of 25% and 70% for PPy(PSS) and PPy(ClO_4_), respectively, compared to the initial experiments. These losses, however, were primarily observed after 40 cycles, coinciding with deviations in the *Q*−/*Q*+ ratio, which steadily increased throughout the process. Consequently, the SRC value declined, while the EC value rose alongside the *Q*−/*Q*+ imbalance.

**Fig. 5:**
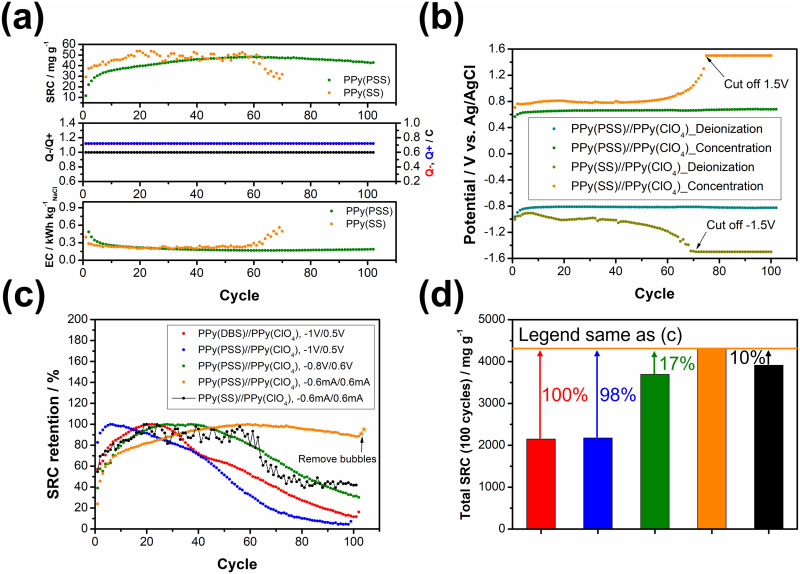
(a) Comparisons between the salt removal capacity (SRC) profile, *Q*−/*Q*+ value, and energy consumption (EC) profile for PPy(PSS)//PPy(ClO_4_) and PPy(SS)//PPy(ClO_4_) with deionization, concentration = −0.6 mA/0.6 mA, 20 min/20 min. (b) The potential profile during the operation in (a). (c) The retention comparison of all systems. (d) Accumulated SRC after 100 cycles for all systems.

While the improved *Q*−/*Q*+ value enhances the overall performance of the system, it still suffers from a breakdown in balance after multiple cycles of operation. This issue stems primarily from the operation mode itself: the constant potential operation method. Since adjusting either part (deionization or concentration) of the potential impacts both aspects of the operation, achieving a perfectly balanced situation through constant potential methods proves to be challenging. Even a minor charge difference between the two processes can lead to significant imbalance after accumulation through prolonged operation. Initially, the *Q*−/*Q*+ ratio ranged from 0.98 to 0.99, but even this slight discrepancy could trigger some minor side reactions, ultimately resulting in electrode degradation. This degradation led to a significant imbalance in the *Q*−/*Q*+ ratio, reaching 1.2 after 100 cycles of operation. The easiest way to achieve perfectly balanced charges is by conducting experiments using the constant current method. By ensuring equal durations for deionization and concentration processes with the same applied current, we attained a system with ideal balance. Fig. 4(d) illustrates the results of the PPy(PSS)//PPy(ClO_4_) system employing ±0.6 mA for both processes over 20 minutes, resulting in 0.72C applied for both deionization and concentration processes throughout the operation. The potential profile of this system is provided in Fig. 5(b), and the values were very close to the parameters of −0.8 V/0.6 V we used in the previous setup but with more flexibility to balance the charges. In this setup, the SRC and SRR remains high at 48 mg g^−1^ and 2.4 mg g^−1^ min^−1^, respectively, with SRC retention at 90% after 100 cycles of operation (Table S2, ESI†), and the EC value significantly lower compared to all four tests (0.17 kW h kg_NaCl_^−1^). Post-cycling CV analysis of both electrodes in Fig. S6(g) and (h) (ESI†) reveals only slight or no decrease in *C*_P_ (PPy(PSS): −17%, PPy(ClO_4_): +3%). After completing the cycling test, small bubbles were generated primarily from dissolved gas in the water source during the process and adhered to the conductivity sensor. This could potentially impact the accuracy of the SRC results. To address this, two additional cycles were conducted under the same conditions after removing these bubbles. The conductivity profile revealed SRC values of 44 mg g^−1^ and 46 mg g^−1^ for these cycles, indicating a minimal 4% loss of SRC and underscoring the superior stability of this system (Fig. S7, ESI†).

To further illustrate the strategies outlined in the previous paragraph, sodium styrene sulfonate (SS) was used as another dopant and compared with the PPy(PSS) electrode. While SS has a similar charge/*M*_W_ ratio compared to PSS, it is much smaller and less bulky. Fig. 5(a) illustrates the performance difference between the PPy(PSS)//PPy(ClO_4_) and PPy(SS)//PPy(ClO_4_) systems. Interestingly, the system with PPy(SS) exhibits a slightly higher SRC compared to the one with PPy(PSS). This difference is attributable to the lower doping amount of PPy(PSS) in comparison with PPy(SS), as observed in Fig. S2 (ESI†),^22^ accompanied with the higher potential profile of PPy(SS) during the constant current operation, resulting in a higher SRC value. The EC profile also shows similar results for these two systems. However, as shown in Fig. 5(b), the potential profile of PPy(SS)//PPy(ClO_4_) starts to rise from cycle 60 and eventually reaches the limit set to protect the system (±1.5 V) by cycle 70. The rising potential once again confirms the occurrence of doping leakage during the deionization process. As SS is even smaller than DBS, these dopants tend to leave the polymer backbone during operation. This departure of dopants further diminishes the specific capacitance of the electrode, consequently increasing the cell voltage and eventually reaching the imposed limitation. The post-cycling CV analysis of PPy(SS) indicates a significant decrease of 89% in specific capacitance, as depicted in Fig. S8 (ESI†). Furthermore, after the cycling tests, the solution was subjected to ICP-OES analysis to measure the sulfate concentration. Since sulfate only appears on the dopants in all systems, changes in its concentration can serve as an indicator of dopant leakage. The system with PPy(PSS) exhibited the lowest concentration of sulfate at 1.7 ppm, while the systems with PPy(SS) and PPy(DBS) exhibited concentrations of 7.4 ppm and 6.7 ppm, respectively (Fig. S9, ESI†).

Fig. 5(c) illustrates the stability comparison of all five systems. In the systems with unbalanced charges, such as PPy(DBS)//PPy(ClO_4_) and PPy(PSS)//PPy(ClO_4_) (depicted in red and blue, respectively), the SRC exhibits early-stage decreases due to electrode damage caused by dopant leakage or irreversible side reactions. When adjusting the working potential to a more balanced situation (depicted in green), the system demonstrates much higher stability but still suffers from late-stage degradation. Only by implementing the strategies we provided above—dopant engineering and balanced charges during operation—the system can achieve remarkable stability, with over 95% retention of SRC when using PSS as the dopant and employing the constant current operation method. The last system, with SS as the dopant (depicted in black), further emphasizes the importance of dopant engineering, as the system still degrades even with perfect charge balance due to dopant leakage. Fig. 5(d) presents the accumulated SRC value after 100 cycles. With the appropriate dopant design and well-balanced charges, the SRC value can almost double from around 2000 mg g^−1^ to over 4000 mg g^−1^. It is worth noting that the difference between the PPy(PSS)//PPy(ClO_4_) system with −0.6 mA/0.6 mA and other systems can further increase, as this system retains around 95% of its SRC, while the stability of other systems remains below 50%. These findings substantiate our initial assumption that through dopant engineering and finely tuned operational parameters, significant enhancements in SRC, EC, and stability can be simultaneously attained, thus amplifying the potential of the full-polymer system.

### 3.3. Post-cycling characterization

**Fig. 6:**
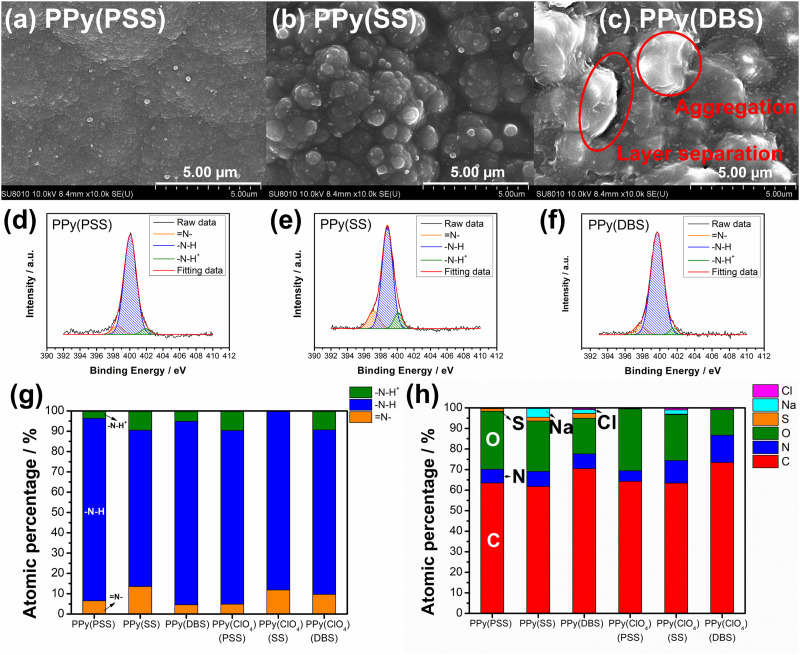
(a)–(c) SEM surface morphologies under 10k magnification and (d)–(f) HRXPS N 1s core level spectra for (a) and (d) PPy(PSS), (b) and (e) PPy(SS), and (c) and (f) PPy(DBS) after the cycling test. (g) The atomic percentages of different nitrogen species and (h) the atomic percentages of S, N, O, C, and Cl on the surface for six PPy materials.

To further investigate the degradation mechanism, the electrodes underwent characterization after 100 deionization/concentration cycles. Fig. 6(a)–(c) showcase the surface morphologies of PPy with different dopants as studied with FESEM. Comparing these images with the pristine surface morphologies in Fig. 1, PPy(PSS) exhibits almost no change, with only minor aggregation on the surface. Similarly, the surface morphology of PPy(SS) remains largely unchanged, displaying a similar aggregation structure but with slightly more indistinct boundaries, possibly due to the loss of dopants altering the PPy structure slightly. However, the morphology of PPy(DBS) undergoes significant alterations from its pristine state, with substantial aggregation and the formation of separate layers on its surface. This change can be reasonably attributed to the leakage of large DBS micelle dopants, leading to deterioration of the surface. The surface morphology of PPy(ClO_4_) remains largely consistent across all cases, as depicted in Fig. S10(a)–(c) (ESI†). Fig. 6(d)–(g) and Fig. S10(d)–(f) (ESI†) present the N 1s core level fitting and the atomic percentage of each material from high-resolution XPS analysis. Comparing these results with those in Fig. 2, it is evident that the percentage of positively charged nitrogen structure (–N–H^+^ bond) decreases in all cases. This decrease can be attributed to two reasons. The primary reason is that in comparison with the cycled PPy films, the pristine PPy films are under the higher mean oxidation state during the anodic polymerization, resulting in a higher density of positively charged species. In addition, every pristine PPy film is believed to be uniform in the positively charged nitrogen structure in the whole polymer matrix since the anodic polymerization is under a constant-current mode (*i.e.*, constant rate of PPy formation). This idea is supported by the linear dependence of PPy mass on the synthesis charged in Fig. S2(a) (ESI†). The minor factor is the loss of dopants during the repeated deionization/concentration cycles, which necessitates maintaining charge neutrality within the structure. This may lead to a potential decrease in the demand for positively charged nitrogen and a transformation into neutral nitrogen (–N–H bonds) or imine nitrogen (=N– bonds) in the polymer backbone.^22^Fig. 6(h) illustrates the surface element composition after cycling. The corresponding doping ratios of PPy(PSS), PPy(SS), and PPy(DBS) are 0.2, 0.25, and 0.33, respectively. Comparing these values with the pristine doping ratios of 0.46, 0.45, and 0.73, respectively, reveals dopant losses at the electrode surface of 57%, 44%, and 55%, respectively. Remarkably, this result contradicts the evidence of the sulfur content in the solution obtained from the ICP-OES analysis (Fig. S9, ESI†). Therefore, a more detailed model for the dopant leakage mechanism can be proposed on the basis of these results. For the smallest dopant, PPy(SS) demonstrates the highest concentration of sulfur in the solution but with the smallest loss of sulfur on the surface. This indicates that during the cycling process, not only does the surface dopant leach into the solution, but also the dopant within the bulk PPy backbone migrates to the surface and eventually leaches into the solution. Furthermore, PPy(DBS) exhibits similar trends, wherein the larger size of the dopants results in more significant dopant loss on the surface and slightly less within the bulk structure. Conversely, PPy(PSS) shows the highest dopant loss on the surface but the lowest sulfur concentration in the solution. This observation suggests that PPy(PSS) only loses some of the dopants that are not effectively trapped on the surface. The remaining dopants within the bulk structure remain integrated into the polymer backbone throughout the cycling test, thus contributing to the excellent stability of the system.

### 3.4. Degradation mechanism

**Fig. 7:**
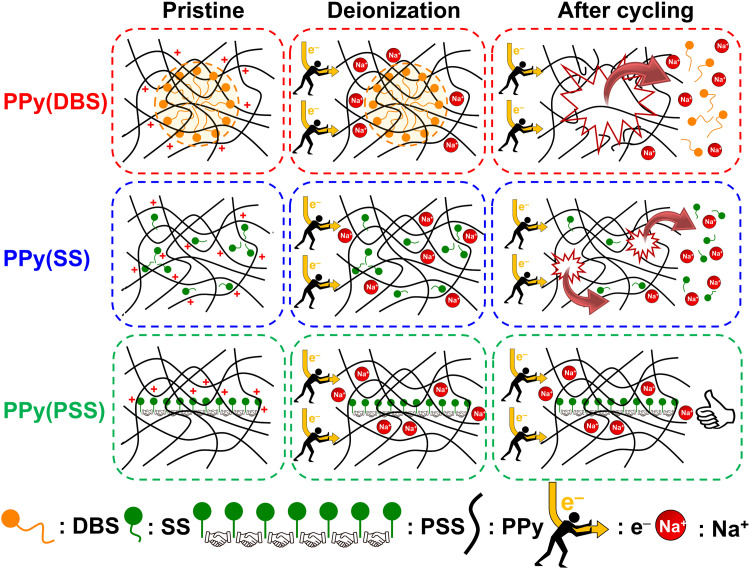
Schematic of the degradation mechanism of PPy(DBS) with large micelles formation, PPy(SS) with no micelle formation or incomplete micelle-like structures formation, and PPy(PSS) with no micelle formation.

**Fig. 8:**
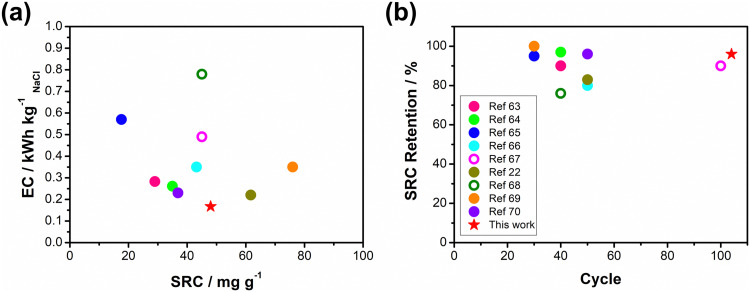
(a) Comparisons of SRC and EC, (b) cycle life and SRC retention between this work and various recently proposed conducting polymer-based or conducting polymer-derived systems.

Based on the results obtained in previous sections, we propose a detailed degradation mechanism for the full-polymer ECDI system, as illustrated in Fig. 7. During the electro-polymerization process, DBS forms micelles in the electrolyte and becomes integrated into the polymer backbone. Conversely, SS is trapped in the polymer backbone without forming a micellar structure (or forming incomplete micelle-like structures) due to the short carbon chain on the SS unit. Similarly, the long PSS chain is incorporated into the PPy film without forming a micellar structure (Fig. 7, pristine). Note that the pristine PPy was in a highly oxidized state that carries positive charges on the polymer chain to compensate the negative charges on the dopants. The different sizes of these micelles (dopants) result in different *d*-spacings within this polymer thin film (Fig. S4, ESI†). During the deionization process, electrons flow from the PPy(ClO_4_) side to the electrode through the external circuit. These electrons neutralize the positive charges present on the polymer chain, rendering the polymer chain charge neutral. This creates a negatively charged environment within the PPy structure due to the presence of dopants. According to the charge compensation mechanism, sodium ions migrate and are trapped inside the polymer backbone to maintain charge neutrality (Fig. 7, deionization). However, these electrons not only attract sodium ions but also provide an electrostatic driving force for pushing out anions from the polymer backbone, *i.e.*, the dopants. Even though SS and DBS are relatively larger in size than ClO_4_^−^ and Cl^−^, they are still subject to this driving force and slowly migrate out of the structure. For SS and DBS, dopant leakage occurs both on the surface and within the bulk structure, leading to structural deterioration of PPy. Moreover, the loss of dopants represents a deficiency in negative charges, resulting in the degradation of ion removal ability (Fig. 7, after cycling). Through appropriate dopant engineering, PSS provides the highest charge density (similar to SS), but with an extremely long and bulky structure, making it very difficult to leak from the polymer backbone during prolonged cycling operations. This characteristic maintains the structural integrity and retains negative charges within the polymer structure, making it an extremely stable positive electrode material for the ECDI system. Furthermore, by optimizing the operating conditions to balance the charges between deionization and concentration processes, the driving force that pushes away the dopant can be minimized. As a result, an ECDI system (PPy(PSS)//PPy(ClO_4_)) with an SRC of 48 mg g^−1^, the lowest EC of 0.167 kW h kg_NaCl_^−1^, an average EC of 0.194 kW h kg_NaCl_^−1^, and 96% SRC retention after 104 cycles of operation was achieved. This system overcomes the common disadvantage of poor stability in PPy-based systems and also provides a high SRC value and low EC value through dopant engineering and parameter optimization, making this full-polymer system even more promising. Fig. 8 (details provided in Table S4, ESI†) compares our designed system with other recently proposed conducting polymer-based or conducting polymer-derived systems.^22,63–70^ Our system demonstrates ultra-low energy consumption coupled with a comparable SRC. Moreover, our system exhibits the best stability performance, operating for most cycles with negligible degradation of SRC. Remarkably, our membrane-free system achieves this level of stability, marking a significant breakthrough. Based on the evidence presented in this study, the PPy(PSS)//PPy(ClO_4_) system has proven to be a potential candidate for future water purification applications. Furthermore, the guidelines and mechanisms elucidated herein can enhance the performance of ICP-based systems in various electrochemistry fields.

## 4. Conclusions

Two strategies have been proposed to enhance the overall performance (SRC, EC, and stability) of a full-conducting polymer ECDI system. Firstly, through meticulous dopant engineering, dopants with high charge density and structural bulkiness can contribute to a high SRC value and substantially reduce dopant leakage issues. Secondly, by balancing the charges between the deionization and concentration processes, the stability of electrodes can be further improved. Consequently, the PPy(PSS)//PPy(ClO_4_) system operated under the constant current method demonstrated a high SRC of 48 mg g^−1^, an ultra-low EC of 0.167 kW h kg_NaCl_^−1^, and remarkable stability with 96% SRC retention after 104 cycles of operation. Furthermore, the leakage of dopants during the deionization and ion-concentration cycling is the main reason responsible for the SRC decline resulting from PPy degradation, supported by the pristine and post-cycling analyses. During the repeated redox cycling of PPy(DBS) and PPy(SS), besides cation capturing, the DBS and SS dopants are electrostatically forced to migrate from the bulk of the polymer backbone to the surface of the electrode and eventually leak into the solution. This migration process can lead to severe loss of active sites and deterioration of the polymer structure. Conversely, PSS is well-trapped in the bulk structure, with only a small portion of PSS on the surface that is not well-trapped leaving during the cycling test, resulting in excellent stability. These studies provide valuable insights for designing full-polymer ECDI systems and other conducting polymer-based electrochemical systems in the future.

## Author contributions

Yi-Heng Tu: conceptualization, methodology, validation, formal analysis, investigation, data curation, writing – original draft preparation, writing – review & editing, visualization. Hung-Yi Huang: validation, formal analysis, investigation, data curation. Yu-Hsiang Yang: writing – review & editing. Louis C. P. M. de Smet: conceptualization, methodology, validation, writing – original draft preparation, writing – review & editing, supervision, project administration. Chi-Chang Hu: conceptualization, methodology, validation, writing – original draft preparation, writing – review & editing, supervision, project administration.

## Data availability

The data that support the findings of this study are available in the ESI.†

## Conflicts of interest

There are no conflicts of interest to declare.

## Supplementary Material

MH-011-D4MH00494A-s001
