# Supplementary material for: A highly stable full-polymer electrochemical deionization system: dopant engineering & mechanism study
Source: Mater Horiz. 2024 Jun 18;11(16):3792–804. doi: 10.1039/d4mh00494a (PMC11318517; doi:10.1039/d4mh00494a)
Supplement: MH-011-D4MH00494A-s001 [file MH-011-D4MH00494A-s001.pdf]

Electronic Supplementary Material (ESI) for Materials Horizons.
This journal is © The Royal Society of Chemistry 2024

Supporting Information

Highly Stable Full-Polymer Electrochemical Deionization System: Dopant Engineering & Mechanism Study

Yi-Heng Tu[a,b], Hung-Yi Huang[a], Yu-Hsiang Yang[a], Louis C. P. M. de Smet*[,b] , Chi-Chang Hu*[,a]

[a] Department of Chemical Engineering
National Tsing Hua University
Hsin-Chu 300044, Taiwan

[b]Advanced Interfaces & Materials
Laboratory of Organic Chemistry
Wageningen University
Stippeneng 4, Wageningen 6708 WE, the Netherlands

This supporting information consists of 16 pages, including 10 figures and 4 tables.

Section 1. Procedure for preparing titanium current collectors:

The titanium sheets, from Raysen Titanium Industry Co. (Taiwan), with geometric size of 7 cm × 1 cm were immersed in a beaker containing 6 M HCl. The beaker was heated to 80°C and kept at this temperature for 30 min. Afterward, the pickled sheets were thoroughly rinsed with deionized water to remove any surface grease, followed by ultrasonic agitation for comprehensive cleaning. The titanium sheets were immersed in the RuO_2 solution (0.3 mg/mL in DI water) and dried in an oven at 80°C for three repetitions. The pretreated titanium sheets were annealed at 250°C for 2.5 h, resulting in a thin layer of RuO_2 forming on the substrate. The RuO_2 layer simultaneously acts as a protective layer against substrate oxidation and improves the coating uniformity of PPy, which provides no ion removal capacity.

Experimental Setup

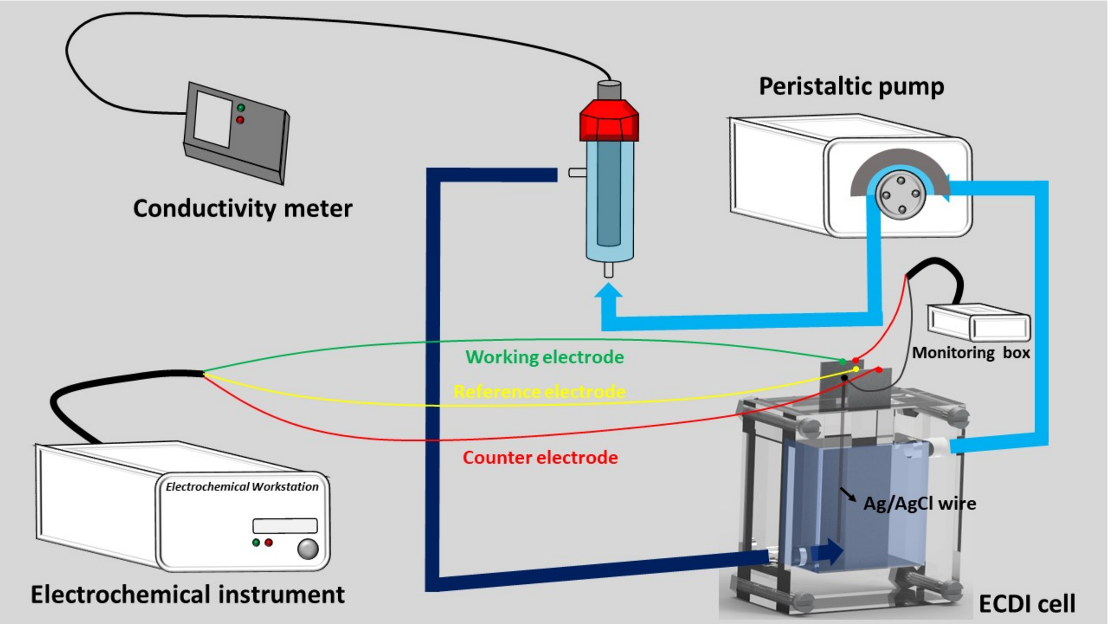

Figure S1. The setup of the desalination test.

Section 2. Characterization

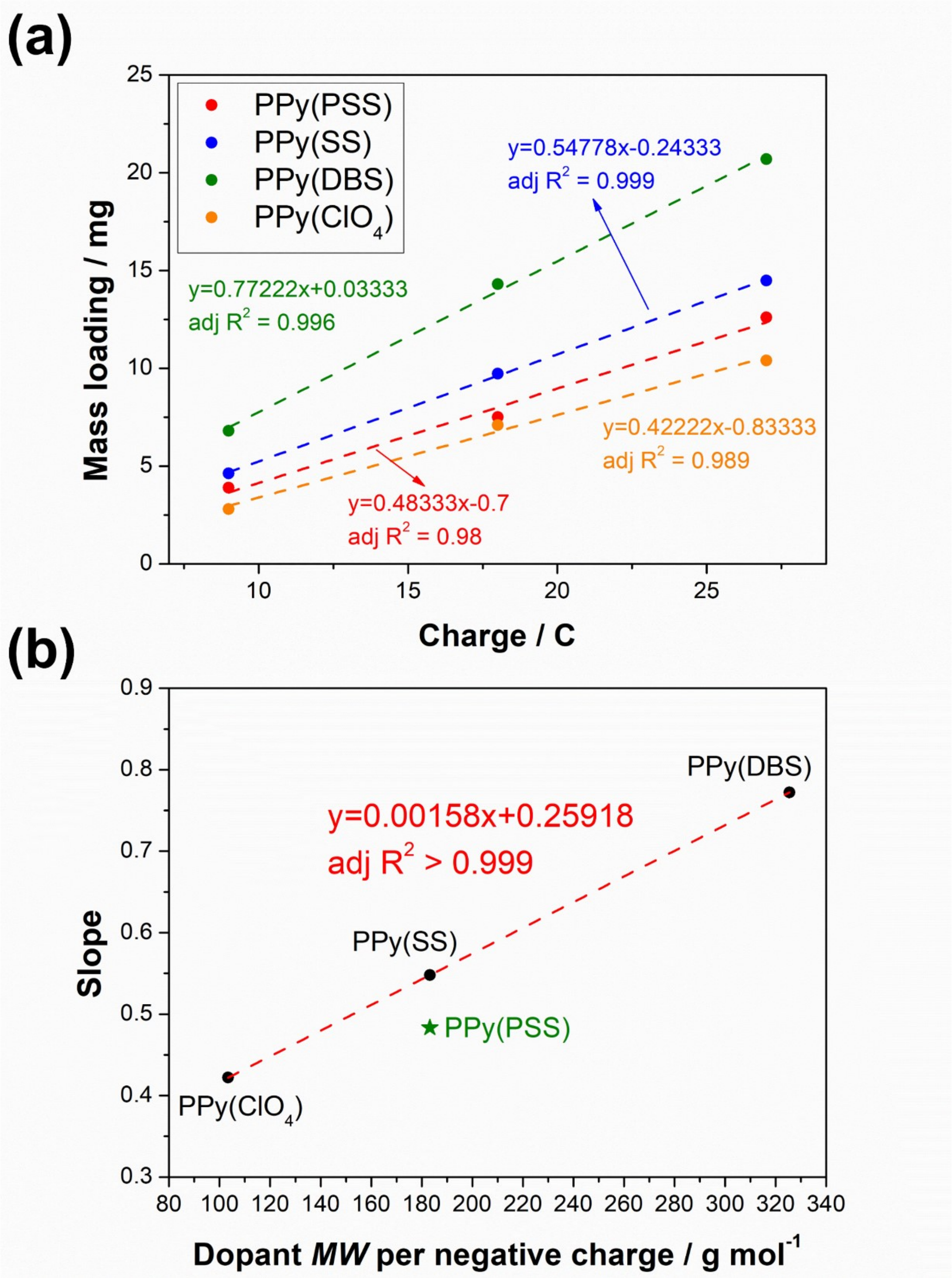

Figure S2. (a) Calibration curves of mass loading vs. charge, (b) relation between slope in Figure S2(a) and molecular weight of dopant per negative charge for PPy(PSS), PPy(SS), PPy(PSS), and PPy(ClO_4).

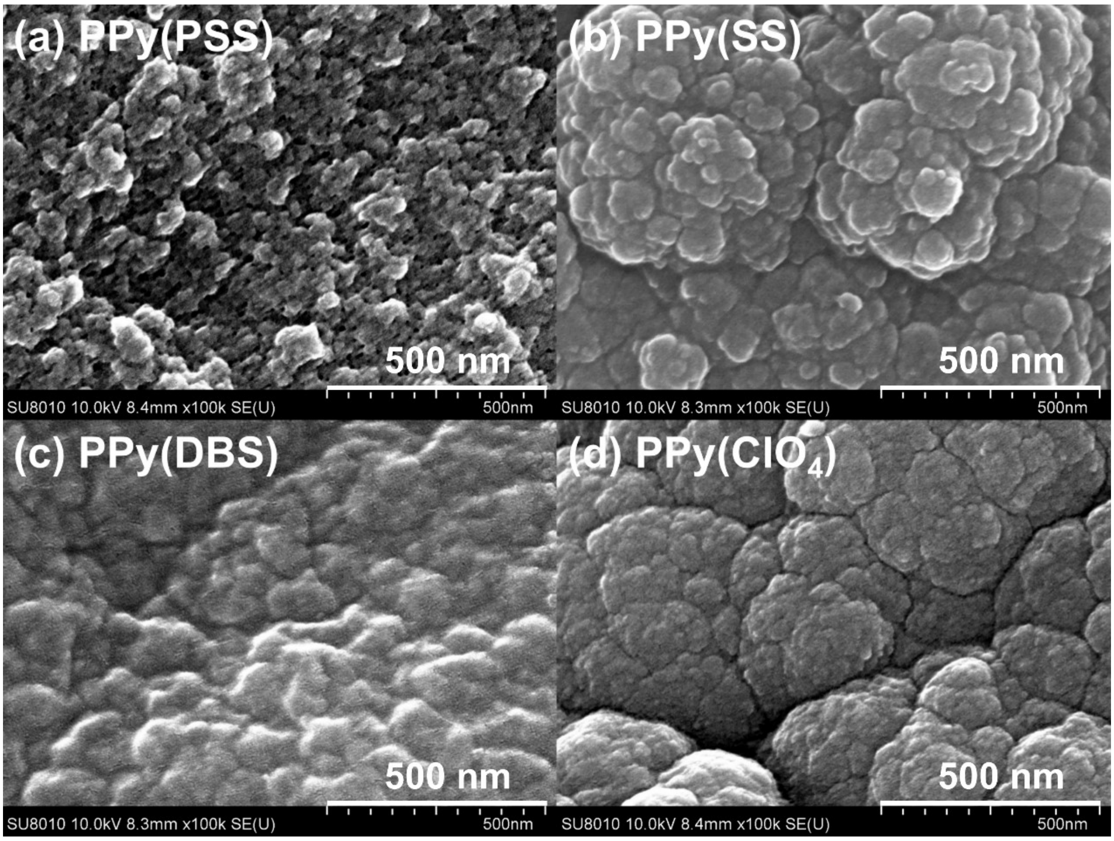

Figure S3. SEM photographs revealing the surface morphologies of (a) PPy(PSS), (b) PPy(SS), (c) PPy(DBS), and (d) PPy(ClO_4) under 100k magnification.

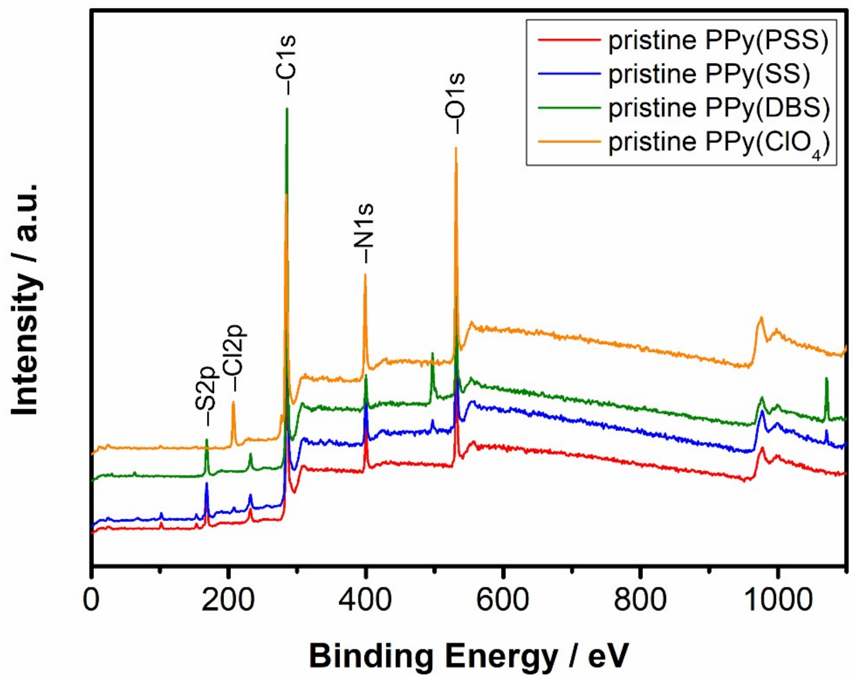

Figure S4. XPS element survey spectra of pristine PPy(PSS), PPy(SS), PPy(DBS), and $PPy(ClO_4)$.

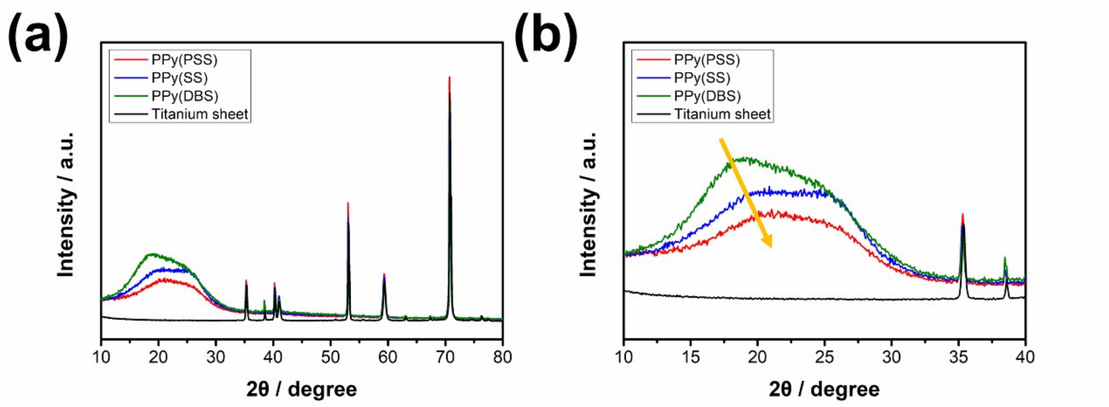

Figure S5. XRD analysis of Ti sheet, pristine PPy(PSS), PPy(SS), and PPy(DBS). (a) Full patterns and (b) patterns with 2θ from 10 to 40°.

Figure S5 shows the typical XRD patterns of various PPy films on the titanium substrates after synthesis. The black line indicates the XRD pattern of a titanium substrate, which most of the peak scatters between 35° to 80°. The responses of the PPy film can be mainly observed between 10° to 30° (2θ). However, due to the poor crystalline structure of PPy, the peaks display in a wide shape.

Table S1. Specific capacitance of PPy(PSS), PPy(SS), PPy(DBS), and PPy(ClO_4) in 10 mM NaCl solution with a potential window of –1.2 V~0.8 V (*vs*. Ag/AgCl) at a scan rate of 2 mV·s^{-1}.

(F/g)	PPy(PSS)	PPy(SS)	PPy(DBS)	PPy(ClO_4)
Specific capacitance (C_P)	91.4	52.2	28.8	62.2

The specific capacitance (C_P) was calculated through the following equation:

$$C_p = \frac{A}{2mk\Delta V} \tag{S1}$$

where A is the area of the CV curve within a certain potential window, m indicates the mass of the electro-active material, k represents the scan rate, and ΔV is the potential window, which is 2 V (–1.2 V~0.8 V) in this case.

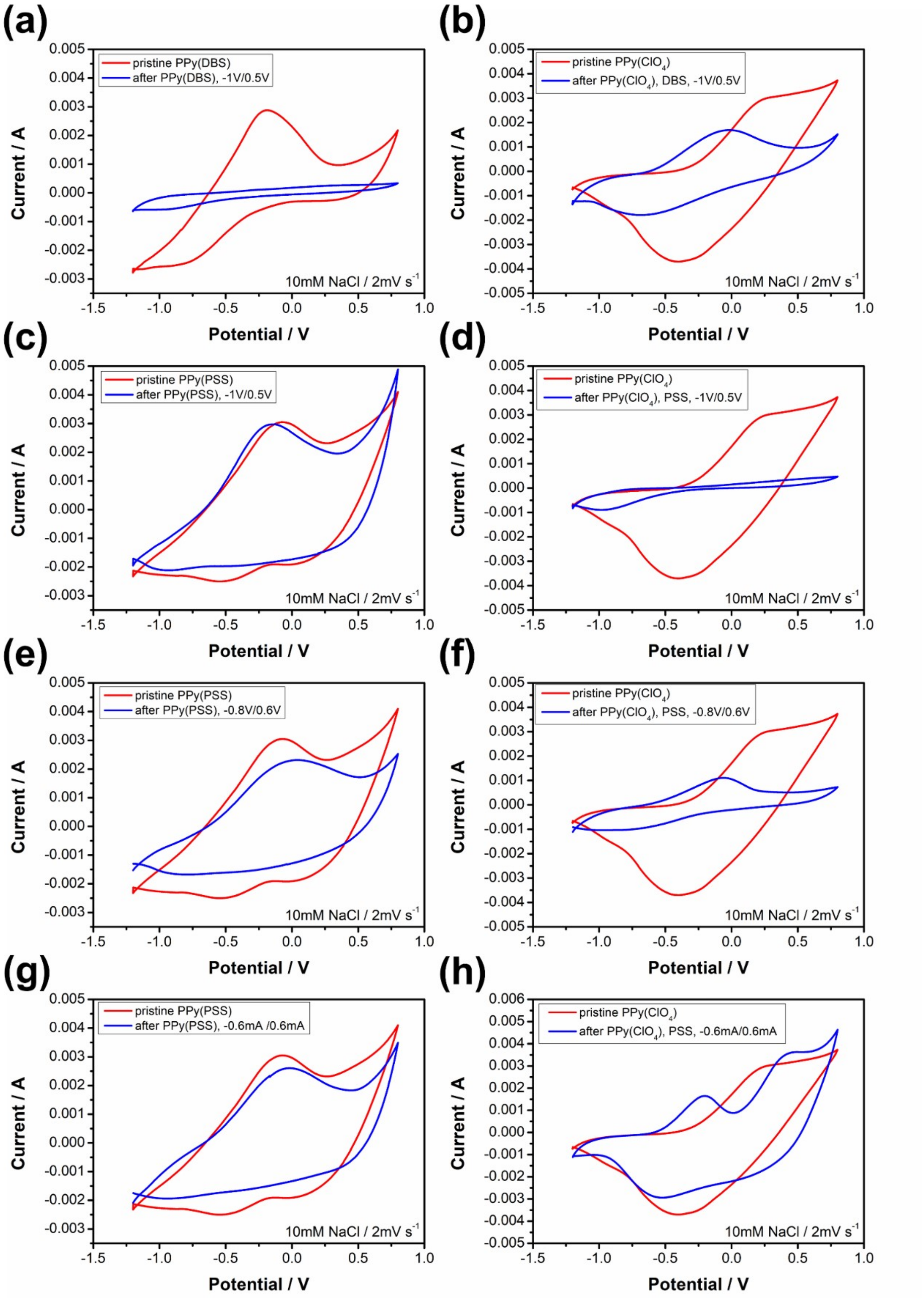

Figure S6. Cyclic voltammograms (CVs) of (a) PPy(DBS), (b, d, f, h) PPy(ClO_4), and (c, e, g) PPy(PSS) before (red) and after (blue) cycling. The PPy(DBS)//PPy(ClO_4) and PPy(PSS)//PPy(ClO_4) cells were cycled with (a-d) –1 V/0.5 V, 20 min/20 min and (e, f) –0.8 V/0.6 V, 20 min/20 min, and (g, h) –0.6 mA/0.6 mA, 20 min/20 min.

Table S2. Detailed values in **Figure 4.**

	Method	Time (min)	Q–/Q+ at cy10	SRC ($mg \cdot g^{-1}$)	Total SRC 100cy ($mg \cdot g^{-1}$)
PPy(DBS)// PPy(ClO_4)	–1V/ 0.5V	20/20	1.21	36.4 ± 0.1	2150.35
PPy(PSS)// PPy(ClO_4)	–1V/ 0.5V	20/20	1.16	42.9 ± 0.3	2174.81
PPy(PSS)// PPy(ClO_4)	–0.8V/ 0.6V	20/20	0.98	49.7 ± 0.1	3692.1
PPy(PSS)// PPy(ClO_4)	–0.6mA/ 0.6mA	20/20	1	48.1 ± 0.1	4311.12
Continue	SRC retention at 40cy	SRC retention at 100cy	Lowest EC ($kWh \cdot kg^{-1}{}_{NaCl}$)	Avg. EC^{+} ($kWh \cdot kg^{-1}{}_{NaCl}$)	
PPy(DBS)// PPy(ClO_4)	72.3%	11.5%	0.438 ± 0.01	0.576	
PPy(PSS)// PPy(ClO_4)	70.8%	5%	0.609 ± 0.05	0.817	
PPy(PSS)// PPy(ClO_4)	99.2%	31.2%	0.429 ± 0.00	0.495	
PPy(PSS)// PPy(ClO_4)	95.2%	89% (96%*)	0.167 ± 0.00	0.194	

*After removing the bubbles in the system.

$^{+}$ Average energy consumption was calculated with the following equation:

$$\frac{Total\ energy\ consumed\ in\ 100\ cycles}{Total\ salt\ being\ removed\ in\ 100\ cycles}$$

Table S3. Specific capacitance (C_P) in **Figure. S6** measured in 10 mM NaCl with a potential window of –1.2 V~0.8 V (*vs*. Ag/AgCl) at a scan rate of 2 mV·s^{-1}

C_P	F/g	(a) (b)	(c) (d)	(e) (f)	(g) (h)
Positive electrode	Pristine	28.8	91.4	91.4	91.4
	After	4.5	91	68.3	75.6
	Difference	–84%	–0.5%	–25.2%	–17%
Negative electrode	Pristine	62.2	62.2	62.2	62.2
	After	35	6.6	18.5	65.6
	Difference	–43%	–89%	–70.2%	+3%

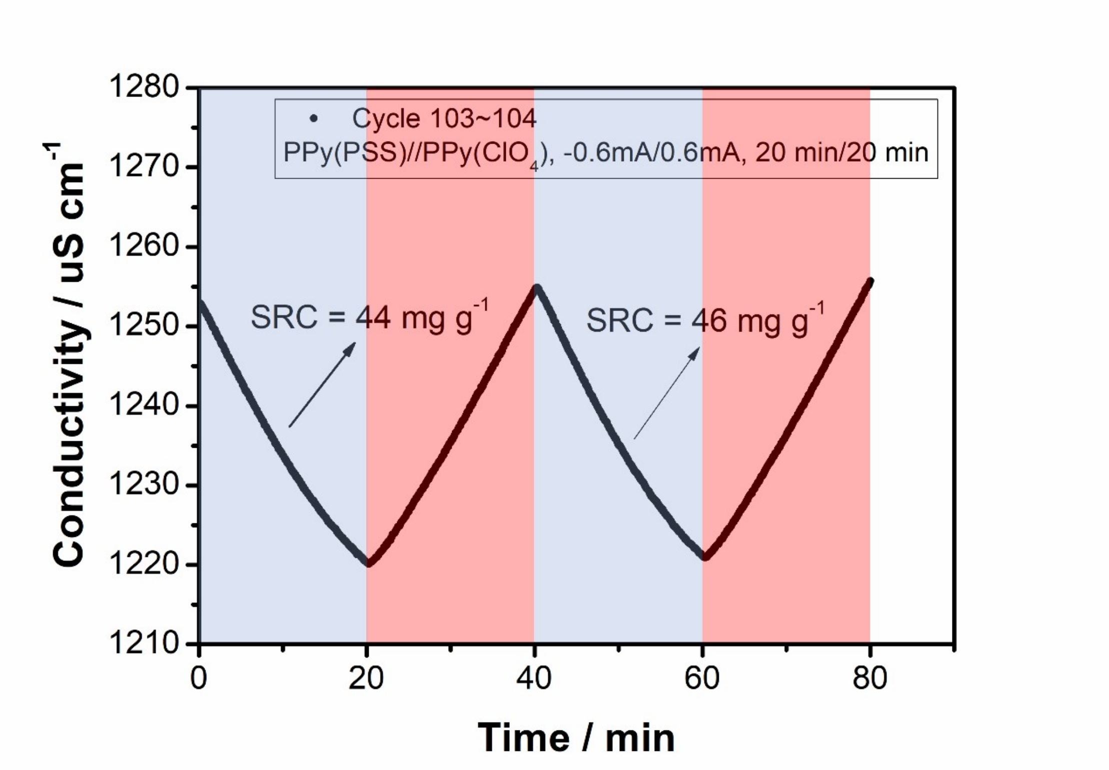

Figure S7. Conductivity-time profiles of the 103rd and 104th cycles for PPy(PSS)//PPy(ClO_4) with deionization/ concentration = –0.6 mA/ 0.6 mA, 20 min/ 20 min.

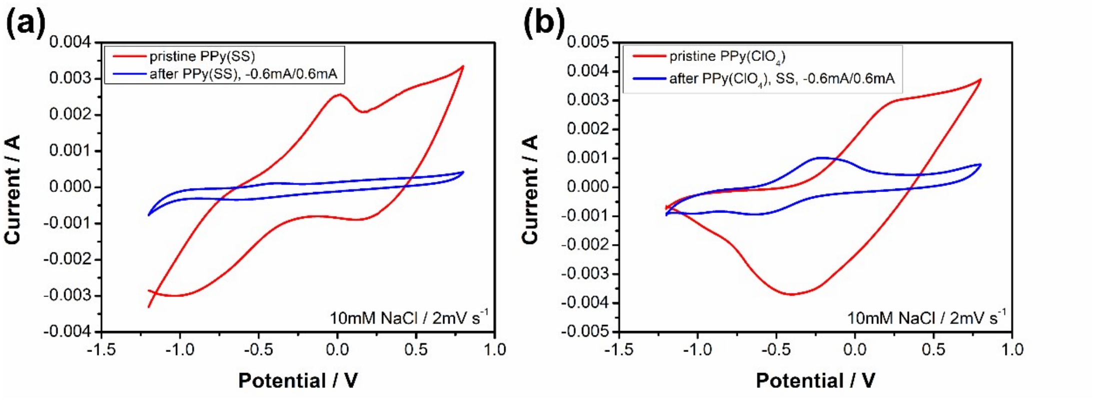

Figure S8. Cyclic voltammograms (CVs) of (a) PPy(SS) and (b) PPy(ClO_4) (1) before and (2) after the PPy(SS)//PPy(ClO_4) cell was cycled with –0.6 mA/0.6 mA, 20 min/20 min.

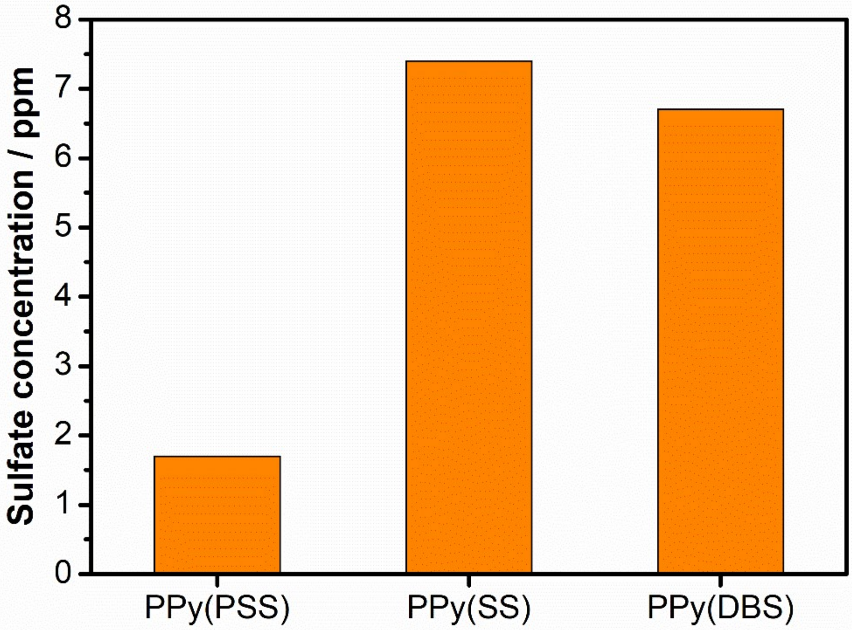

Figure S9. The sulfate concentrations in the 10 mM NaCl solution which was subjected to the 100-cycle test with PPy(PSS), PPy(SS), and PPy(DBS).

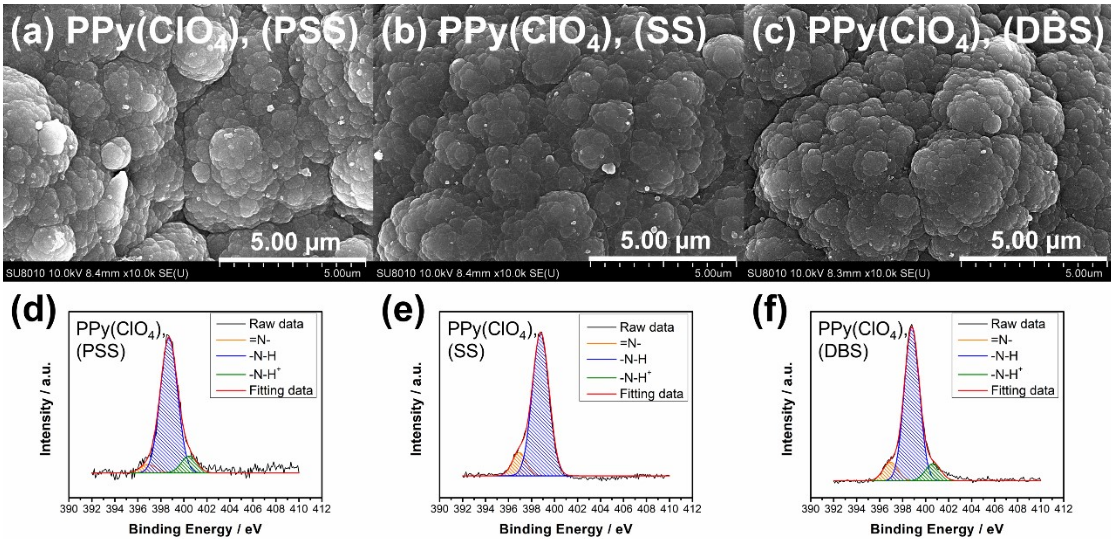

Figure S10. SEM surface morphologies under 10k magnification for (a) PPy(ClO_4) (with PPy(PSS)), (b) PPy(ClO_4) (with PPy(SS)), and (c) PPy(ClO_4) (with PPy(DBS)) after the cycling test. HRXPS N1s core level spectra for (d) PPy(ClO_4) (with PPy(PSS)), (e) PPy(ClO_4) (with PPy(SS)), and (f) PPy(ClO_4) (with PPy(DBS)).

Table S4. Comparisons of SRC, EC, membrane, cycle life, and retention between this work and various recently proposed conducting polymer-based or conducting polymer-derived systems.

Electrode Materials	SRC ($mg \cdot g^{-1}$)	EC ($kWh \cdot kg^{-1}{}_{NaCl}$)	Mem	Cycle	Retention	Ref
AC//Ni,Co-PBA@MXene/PPy	29	0.283	X	40	90%	1
PPy-Cl@MXene //PPy-DBS@MXene	35	0.621	X	40	97%	2
AC//MXene/BC@PPy (MBP)	17.6	0.57	X	30	95%	3
PPy//AC-MnO_2	43.2	0.35	X	50	80%	4
CuHCF@PVA/PPy//AC	45	0.49	O	100	90%	5
PPy-DBS//PPy-ClO_4	61.7	0.22	X	50	83%	6
AC//PPP	45	0.78	O	40	76%	7
S-$Ti_3C_2T_x$/PANI/F-$Ti_3C_2T_x$ // AC	76	0.35	X	30	100%	8
H-NP@PANI//AC	36.9	0.23	X	50	96%	9
PPy(PSS)//PPy(ClO_4)	48.1±0.1	0.123 ± 0.00	X	104	96%	This work

Note: Mem = Membranes

References

1. Y. Cai, W. Zhang, J. Zhao and Y. Wang, *Applied Surface Science*, 2023, **622**, 156926.
2. L. Zhang, Y. Cai, R. Fang, Y. Wang and S. Xu, *Separation and Purification Technology*, 2024, **337**, 126362.
3. W. Xu, C. Tan, A. Wang, S. Hu, L. Deng, S. Boles, K. Sun, B. Li and H. Hu, *ACS Applied Materials & Interfaces*, 2023, **15**, 16266-16276.
4. G. Tan, S. Lu, N. Xu, D. Gao and X. Zhu, *Environmental Science & Technology*, 2020, **54**, 5843-5852.
5. Y. Ren, F. Yu, X.-G. Li, B. Yuliarto, X. Xu, Y. Yamauchi and J. Ma, *Materials Horizons*, 2023, **10**, 3548-3558.
6. H.-Y. Huang, Y.-H. Tu, Y.-H. Yang, Y.-A. Chen, W.-L. Lee, M.-F. Wu, H.-H. Chou and C.-C. Hu, *Journal of Materials Chemistry A*, 2024, **12**, 4312-4324.
7. W. Kong, X. Ge, M. Yang, Q. Zhang, J. Lu, H. Wen, H. Wen, D. Kong, M. Zhang and X. Zhu, *Desalination*, 2023, **553**, 116452.
8. X. Yang, Z. Jia, W. Zhang, G. Ying, Z. Wang, Z. Lu and J. Zhang, *Desalination*, 2022, **535**, 115816.
9. J. Guo, Y. Wang, H. Zhang, Y. Cai and R. Fang, *Desalination*, 2023, **548**, 116305.
